# Aging in Mice Reduces the Ability to Sustain Sleep/Wake States

**DOI:** 10.1371/journal.pone.0081880

**Published:** 2013-12-16

**Authors:** Mathieu E. Wimmer, Justin Rising, Raymond J. Galante, Abraham Wyner, Allan I. Pack, Ted Abel

**Affiliations:** 1 Neuroscience Graduate Group, University of Pennsylvania, Philadelphia, Pennsylvania, United States of America; 2 Department of Biology, University of Pennsylvania, Philadelphia, Pennsylvania, United States of America; 3 Statistics Department, The Wharton School, University of Pennsylvania, Philadelphia, Pennsylvania, United States of America; 4 Center for Sleep and Circadian Neurobiology, University of Pennsylvania, Philadelphia, Pennsylvania, United States of America; 5 Division of Sleep Medicine, Department of Medicine, University of Pennsylvania, Philadelphia, Pennsylvania, United States of America; Univ. Kentucky, United States of America

## Abstract

One of the most significant problems facing older individuals is difficulty staying asleep at night and awake during the day. Understanding the mechanisms by which the regulation of sleep/wake goes awry with age is a critical step in identifying novel therapeutic strategies to improve quality of life for the elderly. We measured wake, non-rapid eye movement (NREM) and rapid-eye movement (REM) sleep in young (2–4 months-old) and aged (22–24 months-old) C57BL6/NIA mice. We used both conventional measures (*i.e.*, bout number and bout duration) and an innovative spike-and-slab statistical approach to characterize age-related fragmentation of sleep/wake. The short (spike) and long (slab) components of the spike-and-slab mixture model capture the distribution of bouts for each behavioral state in mice. Using this novel analytical approach, we found that aged animals are less able to sustain long episodes of wakefulness or NREM sleep. Additionally, spectral analysis of EEG recordings revealed that aging slows theta peak frequency, a correlate of arousal. These combined analyses provide a window into the mechanisms underlying the destabilization of long periods of sleep and wake and reduced vigilance that develop with aging.

## Introduction

Life expectancy is on the rise worldwide. Within the US alone, it is estimated that 20% of the population will be over the age of 65 years by 2030 (U.S. Census Estimate). Normal aging produces sleep fragmentation and impairs the ability to sustain wakefulness in humans [1], [2], [3], [4], [5], [6], and rodents [7], [8], [9], [10], [11], [12], [13], [14], [15], [16], [17], [18]. Aging has also been shown to impact the amplitude and timing of circadian biological rhythms [3] and reduce the homeostatic response to sleep loss [9]. Previous studies suggest that alterations in neurotransmitter and receptor levels in brain regions that regulate sleep/wake underlie these age-induced disruptions in sleep. Aged animals show lower extracellular levels of the wake promoting peptide hypocretin (orexin) and reduced expression of hypocretin receptors [19]. Orexinergic and noradrenergic neurons in aged mice show reduced activity during the active phase [13]. These age-related disruptions in signaling may underlie the inability to maintain wakefulness and sleep, as well as the alterations of EEG spectral profile that accompany normal aging. These two hallmarks of aging have been well characterized in humans [3], [6] and rats [12]. However, few studies have investigated the effects of normal aging on sleep in mice [9], [13], and only one recent report [9] examined the impact of aging on the spectral profile of the EEG during sleep. To address changes in sleep with aging in mice to lay the groundwork for future genetic studies, we studied differences in EEG spectral profile and sleep architecture of young and aged C57BL/6 mice, one of the most commonly used stains of mice in genetic and pharmacological studies. The unique architecture of rodent sleep is characterized by the uneven distribution of short and long bouts in each behavioral state [20], [21], [22], rendering average bout duration a poor descriptor of sleep/wake structure. Here, we used an innovative statistical approach [23] that faithfully models both components of each behavioral state and permits the analysis of short and long bouts simultaneously. We hypothesized that aging would impair the ability of mice to sustain the longer bouts of sleep and wake.

## Methods

### Ethics Statement

All animal care and experiments were approved by the Institutional Animal Care and Use Committee of the University of Pennsylvania and conducted in accordance with the National Institute of Health guidelines. Efforts were made to limit the number of animals used in each experiment and to minimize animal suffering using anesthetics and analgesics.

### Animals and surgery

8 young (2–4 months) and 12 old (22–24 months) male C57BL/6NIA mice were obtained from the National Institute of Aging mouse colony. Animals were maintained on a 12 hour light/12 hour dark cycle with lights on (ZT 0) at 7:00 am. Food and water were available *ad libitum.* All animal care and experiments were approved by the Institutional Animal Care and Use Committee of the University of Pennsylvania (permit # 801547) and conducted in accordance with the National Institute of Health guidelines. Animals were implanted with EEG and EMG electrodes under isoflurane anesthesia and all efforts were made to minimize animal suffering. Electrodes were held in place with dental cement (Ketac, 3M, St Paul, MN). Electrodes consisted of Teflon coated wires (Cooner wires, Chatsworth, CA) soldered to gold socket contacts (Plastics One, Roanoke, VA) and pushed into a 6-pin plastic plug (363 plug, Plastics One). The contacts were cemented to the plug using dental cement. Animals were connected to amplifiers using light-weight cables (363, Plastics One) attached to a rotating commutator (SLC6, Plastics One). All recordings were obtained using parietal electrodes (ML ±1.5 mm, AP −1.5 mm from bregma) referenced to an electrode over the cerebellum (1.5 mm posterior of lambda). Mice were allowed to recover from surgery for a minimum of 2 weeks. During the second week of recovery, mice were acclimated to the cables and to the recording chambers.

### EEG recordings and analysis

EEG/EMG signals were sampled at 256 Hertz (Hz) and filtered at 0.5–30 Hz and 1–100 Hz, respectively with 12A5 amplifiers (Astro-Med, West Warwick, RI). Data acquisition and visual scoring was performed using SleepSign software (Kissei Comtec, INC, Japan). EEG/EMG recordings will be stored on the Abel lab server and are available upon request. EEG/EMG recordings were scored in 4-second epochs as wake, NREM, or REM by a trained experimenter blind to age. Epochs containing movement artifacts were included in the state totals and architecture analysis, but excluded from subsequent spectral analysis. Spectral analysis was performed using a fast Fourier transform (FFT; 0.5–20 Hz, 0.25 Hz resolution). Wake EEG spectra were computed during the dark phase, when wakefulness prevails. NREM and REM EEG spectra were calculated during the light phase, when mice are mostly asleep. NREM slow wave activity (SWA) was computed across the 24-hour recording period and SWA was normalized to the last 4 hours of the light phase for each animal as previously described [24].

**Statistics:** Student's t-tests were used to compare wake, NREM and REM sleep levels averaged over 24 hours. Multivariate analysis of variance (MANOVA) was used on the proportion of time spent in each state during the light phase, the dark phase and during the 8×3-hour bins across 24 hours, followed by Tukey studentized range tests to compare the 2 age groups. The same procedure was applied to the number of bouts and the average bout duration to compare age groups. Analyses were conducted using either SPSS (version 17.0) or SAS (version 9.2). Tukey studentized range tests were used to analyze raw EEG spectra for wake, NREM and REM sleep. Student's t-tests were used to analyze theta peak frequency for wake and REM EEG spectra. NREM SWA was analyzed using MANOVA followed by Tukey studentized range tests. SWA during the light period and during the dark phase were analyzed separately. Pearson's correlation was used to examine the relationship between time awake during the dark phase and SWA during the first two hours of the subsequent light period.

### Spike and Slab Statistical Model

A spike-and-slab statistical model was also used to examine sleep/wake microstructure. This mixture model, where short bouts are represented by the spike and long bouts are captured by the slab component was previously described in detail [23]. Briefly, this approach models the sequence of unique sleep/wake states and their duration. The spike and slab formulation is used to generate a set of 12 descriptive summary statistics that can be further distilled into three key parameters: 1) n, the number of bouts of the behavioral state, conditional on the previous state 2) π, the proportion of spikes (short bouts) 3) μ, the average duration of the slabs (long bouts).

## Results

### Aged mice have decreased wakefulness during the active period

**Figure 1:**
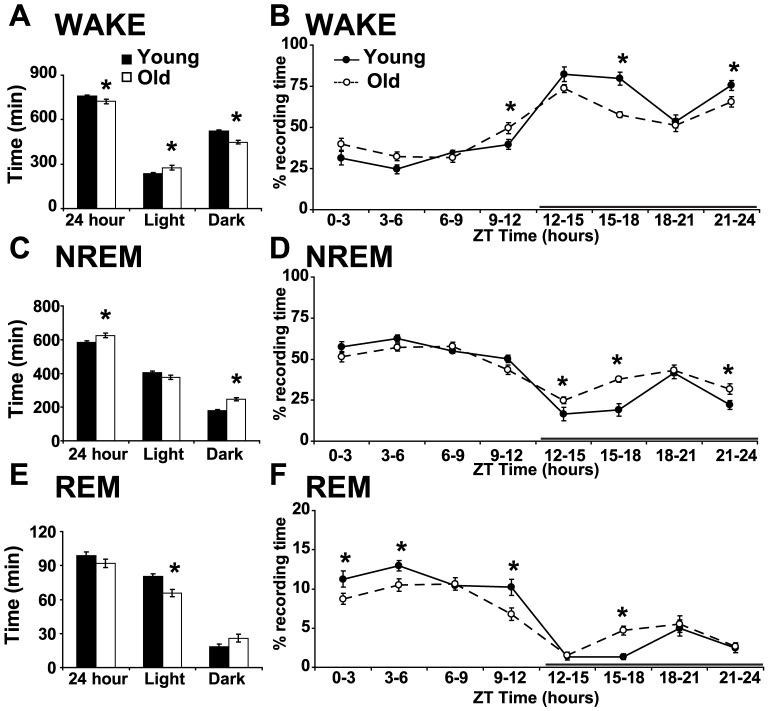
Aged mice show reduced wakefulness during dark phase and decreased REM sleep during light phase. **A**. Total time (min) spent awake for young and old mice over 24 hours (left) and during the light and dark period (right). **B**. Percent time spent awake shown in 8×3-hour bins across light/dark cycle. Solid line represents the dark period **C**. Aged mice show increased NREM sleep during the dark phase. Total time spent in NREM sleep over 24 hours (left) and during the light and dark period (right). **D**. Percent time spent in NREM in 3-hour bins. **E**. Old mice have decreased REM sleep during the rest period. Total time spent in REM sleep for young and old mice over 24 hours (left) and during the light and dark period. **F**. Percent time spent in REM sleep shown in 3-hour bins across light/dark cycle. Mean ± standard error of the mean (s.e.m.), * p<0.05.

We used EEG/EMG recordings from young (2–4 months) and old (22–24 months) mice to quantify wake, NREM and REM sleep. Aged animals showed decreased wakefulness over 24 hours compared to young mice (Satterthwaite t-test, p = 0.039, Fig. 1A). Aged mice spend more time awake during the light period compared to young animals and less time awake during the dark phase, when mice are typically active (F(2,17) = 10.84, p<0.001, Fig. 1A). We also found that age affects the percentage of time spent awake across the light/dark cycle (F(8,11) = 7.4, p = 0.0017, Fig. 1B). We used Tukey studentized range tests to examine the effect of age at 8×3 hour bins across the 24-hour period (Fig. 1B). These tests revealed that aged mice have more wakefulness at the end of the light period and show decreased wake during most of the dark phase (Fig. 1B). Decreased wakefulness in aged mice over 24 hours was accompanied by increased NREM sleep (t-test, p = 0.031). This increase was most obvious during the dark phase (F(2,17) = 12.91, p<0.001, Fig. 1C). Indeed, aged mice showed increased NREM sleep at 3 of the 4 time points during the dark period (F(8,11) = 7.3, p = 0.002, Fig. 1D). REM sleep was overall unchanged when averaged over 24 hours (t-test, p = 0.17, Fig. 1E). However, aged animals showed decreased REM sleep during the light phase (F(2,17) = 6.51, p = 0.008, Fig. 1E). When analyzed in 3 hour bins, aged animals showed decreased REM sleep during most of the light phase and increased REM sleep during one time point of the dark phase (F(8,11) = 4.3, p = 0.014, Fig. 1F).

### Aged mice do not sustain sleep/wake states

**Figure 2:**
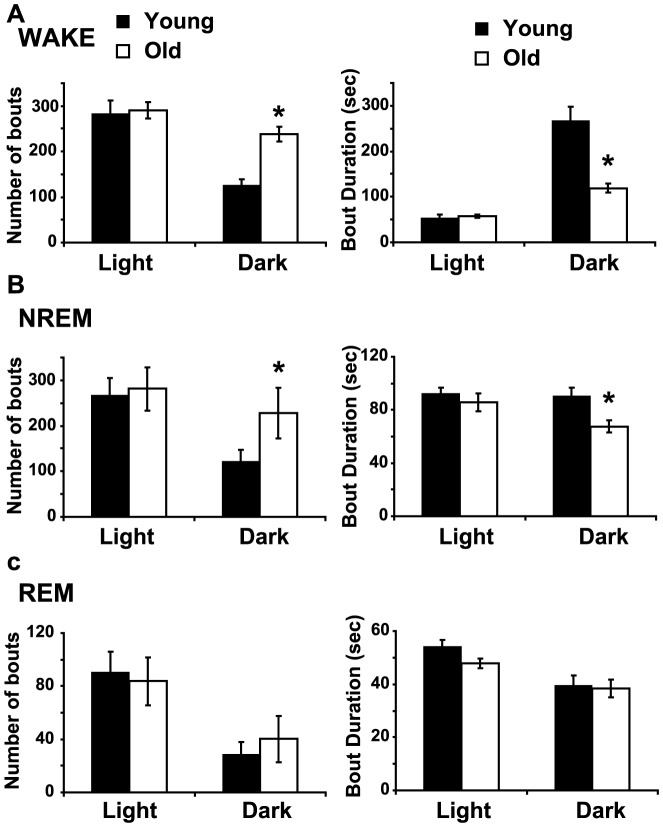
Aging causes fragmentation of wake and NREM sleep. **A**. Number of bouts of wakefulness (left) and average wake bout duration (right) during the light and dark phase. Aged mice show more bouts of wakefulness of shorter average duration during the dark phase. **B**. Aged mice show fragmented NREM sleep during the dark phase. Number of NREM bouts (left) and average NREM bout duration (right) during the light and dark phase. **C**. Number of REM episodes (left) and average REM bout duration during the light and dark period. Mean ± s.e.m., * p<0.05.

**Figure 3:**
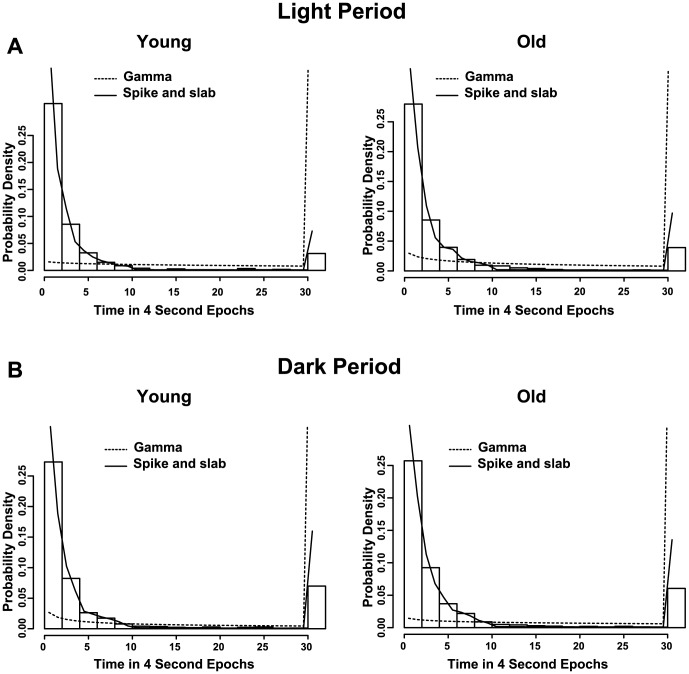
Spike and slab model shows improved fit over a single distribution. Distribution of NREM to Wake bout durations are shown for young and old mice during the light (**A**) and dark period (**B**). The solid line represents the density of the spike and slab model, and the dashed line represents the density of the traditional gamma model. The gamma model assigns too little probability to short bouts and too much probability to long bouts.

**Figure 4:**
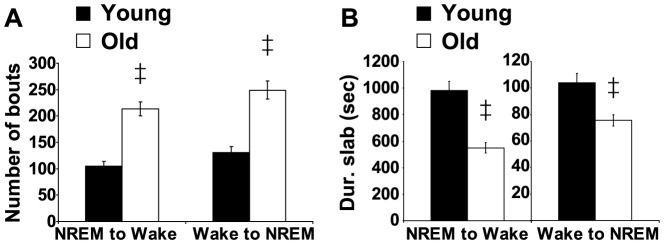
Aged mice are unable to sustain long bouts of wakefulness and NREM sleep. **A**. Number of bouts during the dark phase for young and old mice transitioning from NREM to wake (left) and from wake to NREM (right). Aged mice show an increased number of episodes for both states. **B**. Average duration of slabs (long bouts) for mice transitioning from NREM to wake (left) and wake to NREM (right). The duration of the slabs for both transitions is reduced in aged animals. Mean ± s.e.m., ‡ p<0.00625 (Bonferroni correction).

Next, we examined sleep and wake architecture in young and aged mice using conventional measures (*i.e.*, number of bouts and average bout duration) for each state. Aged mice had more bouts of wakefulness during the dark period compared to young mice (F(2,17) = 14.90, p = 0.0002) and the average duration of wake bouts was shorter in old mice during the dark phase (F(2,17) = 18.07, p<0.0001, Fig. 2A). Similarly, the number of NREM bouts was higher in old mice during the dark period (F(2,17) = 12.31, p = 0.0005) and the average NREM bout duration was lower in aged animals (F(2,17) = 9.7, p = 0.0015, Fig. 2B). The number of REM bouts was not affected by age (F(2,17) = 2.46, p = 0.1151) and neither was the average duration of REM bouts (F(2,17) =  2.14 , p = 0.1484, Fig. 2C). We also used a new spike-and-slab mixture model to compare sleep/wake microstructure in young and aged animals [23]. Typically, bout durations have been modeled as gamma-distributed random variables. However, we found that gamma assigns too little probability to short bouts and too much probability to very long bouts (Fig. 3). The short (spike) and long (slab) components of this model permit an improved fit of the distribution of bout durations in mice (Fig. 3). This was confirmed using BIC and Q-Q plots-based analyses. In addition, we found that the duration of a bout in a given state is dependent upon the previous state of the animals (Table 1). Therefore, each state was analyzed using two models (i.e. wake was subdivided into NREM to wake and REM to wake. Note: REM to NREM transitions were too rare to include in this analysis). The number of NREM to wake episodes and wake to NREM bouts was higher in aged mice (p = 9.35×10^−6^ and p = 6.43×10^−5^, respectively, Fig. 4A). In addition, the average duration of the slab was shorter for both wake and NREM sleep in aged mice (p = 1.53×10^−5^ for NREM to wake and p = 1.67×10^−3^ for wake to NREM, Fig. 4B). Thus, the major difference between young and old mice is in the ability of old mice to sustain long bouts of NREM sleep and long bouts of wakefulness. The architecture of sleep/wake was only disrupted when animals were transitioning between wake and NREM sleep. Aging did not disrupt the architecture of REM sleep during the dark phase (Table 2) or during the light phase (Table 3).

**Table 1:** Bout duration in a given state is conditional on the previous state.

Age	Period	NREM to Wake	REM to Wake	p-value	REM to NREM	Wake to NREM	p-value
Young	Light	2	4	2.09×10^−45^ *	10	14	0.001*
Young	Dark	2	4	6.33×10^−19^ *	4.5	11	–
Old	Light	2	3	1.64×10^−40^ *	9	16	0.002*
Old	Dark	2	5	3.96×10^−24^ *	1	16	0.065

Wake and NREM bout durations were subdivided according to the previous state. Shown are the medians (in 4 second epochs) for each group. Kruskal-Wallis tests were used to compare bout durations for young and old mice during the light and dark period.

indicates p-values that are significant after Bonferonni correction.

**Table 2:** Spike-and-slab analysis, dark period.

Transition	Quantity	Young	Old	p-value
	n	25.88±9.06	36.00±16.10	0.125
NREM to REM	μ	19.22±4.20	20.93±7.52	0.569
	π	0.55±0.16	0.59±0.23	0.7
	n	**104.63±25.06**	**213.67±46.01** *	**9.35×10^−6^**
NREM to Wake	μ	**245.79±48.58**	**137.39±34.54** *	**1.53×10^−5^**
	π	0.79±0.085	0.81±0.04	0.574
	n	25.88±8.86	35.67±16.12	0.136
REM to Wake	μ	363.83±363.224	93.55±52.70	0.019
	π	0.77±0.07	0.80±0.08	0.437
	n	**130.38±31.86**	**249.08±59.11** *	**6.43×10^−5^**
Wake to NREM	μ	**26.02±5.07**	**18.89±3.60** *	**1.67×10^−3^**
	π	0.27±0.09	0.28±0.16	0.794

= number of episodes, μ = average duration of slabs (long bouts, in epochs), π = probability that the animal is in a spike (short bout). Mean ± standard deviation. Spike-and-slab analysis of each state during the dark period for young and old mice. n

p<0.00625 (Bonferroni correction).

**Table 3:** Spike-and-slab analysis, light period.

Transition	Quantity	Young	Old	p-value
	n	84.00±11.28	77.00±16.51	0.311
NREM to REM	μ	21.74±1.54	22.14±3.06	0.734
	π	0.45±0.11	0.54±0.11	0.074
	n	205.38±49.69	224.67±49.65	0.406
NREM to Wake	μ	90.64±21.43	84.91±22.02	0.571
	π	0.89±0.03	0.83±0.06	0.025
	n	80.50±10.39	73.00±16.73	0.275
REM to Wake	μ	66.29±41.66	42.06±17.29	0.086
	π	0.88±0.05	0.86±0.10	0.533
	n	284.88±53.42	297.25±58.10	0.636
Wake to NREM	μ	25.52±4.28	23.73±4.85	0.409
	π	0.22±0.09	0.26±0.12	0.529
	n	4.125±1.126	4.167±2.250	0.962
REM to NREM	μ	12.346±10.995	16.584±18.730	0.572
	π	0.250±0.250	0.310±0.358	0.69

= number of episodes, μ = average duration of slabs (in epochs), π = probability that the animal is in a spike. Mean ± standard deviation. No differences were significant during the light period. Spike-and-slab analysis of each state during the light period for young and old mice. n

### Aged mice show slower theta peak frequency (TPF) and reduced slow wave activity (SWA) at low frequencies (0.5–1.5 Hz)

**Figure 5:**
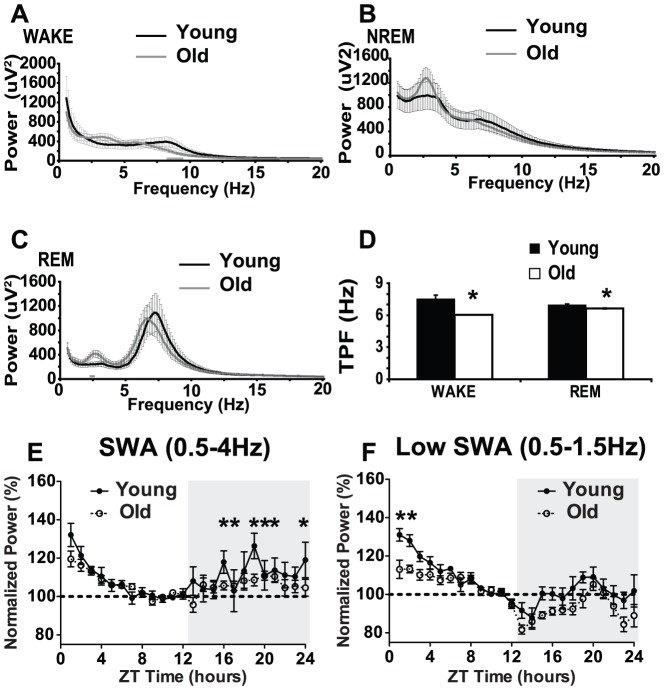
Aging slows theta peak frequency (TPF) and decreases slow wave activity (SWA) at low frequencies (0.5–1.5 Hz). **A**. Wake EEG spectra calculated during the dark period for young and old mice. Aged animals show lower power in the higher frequency range. **B**. EEG spectra for NREM sleep generated during the light period for young and aged mice. Aging did not profoundly affect NREM spectral profile. **C**. EEG spectra of REM sleep computed during the light phase for young and old mice. **D**. Theta peak frequency (TPF) for wake and REM EEG during the dark and light phase, respectively. TPF slowed with aging for both states. **E**. Slow wave activity (0.5–4 Hz, SWA) of NREM sleep for young and old mice expressed relative to the last 4 hours of the light period ( = 100%) for each animal. Grey area denotes the dark period. **F**. Low frequency (0.5–1.5) SWA of NREM sleep young and old mice expressed relative to the last 4 hours of the light period ( = 100%) for each animal. Grey area denotes the dark period. Mean ± s.e.m., gray bar and * indicate p<0.05.

We used fast Fourier transform (FFT) of EEG recordings to examine the EEG spectral profiles during each behavioral state for both age groups. Wake EEG spectra from both young and old animals showed a peak in the theta frequency range but aged mice showed reduced power in the theta range at frequencies between 8.5–9.625 Hz (Tukey Studentized range t-test, Fig. 5A). Interestingly, age did not affect NREM sleep spectra, which showed a characteristic peak in the delta (0.5–4 Hz) frequency range in both young and aged animals (Fig. 5B). Aged mice showed higher power for a small range of frequencies of the REM sleep spectra (2.375–2.625, Fig. 5C). Theta peak frequency (TPF) was determined by recording the frequency at which absolute power was highest in the theta range (6–10 Hz). Aging slowed TPF by about 1.5 Hz from 7.5±0.39 Hz to 6.03±0.02 Hz in the wake EEG spectra (Satterthwaite t-test, p = 0.0072, Fig. 5D). Similarly, TPF was lower by about 0.34 Hz in aged mice (6.63±0.05 Hz) compared to young (6.97±0.09 Hz) in the REM spectra (t-test, p = 0.0026, Fig. 5D). The height of the peak in the theta range was not changed by aging during wake or REM sleep. Slow wave activity (SWA), the spectral power of the EEG in the 0.5–4 Hz range during NREM sleep, is the best characterized marker of sleep intensity and changes in response to sleep loss [25]. SWA (0.5–4 Hz) decreased over the course of the light phase (F(11,176) = 22.59, p<0.0001, Fig. 5E), and SWA (0.5–4 Hz) was not different between young and aged mice (F(11,176) = 1.72, p = 0.1406, Fig. 5E). These results suggest that sleep pressure is discharged similarly during the rest period in young and aged mice. During the dark period, increased SWA was more pronounced in young animals compared to aged mice (F(11,99) = 5.58, p = 0.0009, Fig. 5E). Time spent awake during the dark phase was not correlated with SWA (0.5–4 Hz) during the first two hours of the subsequent light phase (Pearson's, R^2^ = 0.14, p = 0.54).

SWA in lower frequencies (0.5–1.5 Hz) is sensitive to changes in arousal and exploratory behavior during the preceding active period [26], [27]. Thus, changes in low frequency SWA (0.5–1.5 Hz) is indicative of altered vigilance and exploration during wakefulness. We found that low frequency SWA (0.5–1.5 Hz) decreases over the course of the light phase (F(11,165) = 16.75, p<0.0001, Fig. 5F) and that low frequency SWA is lower during the first two hours of the light phase in aged mice compared to young animals (F(11,165) = 2.96, p = 0.0038, Fig. 5F). During the dark phase, age did not affect low frequency SWA (F(11,99) = 0.87, p = 0.5086, Fig. 5F). Interestingly, time awake during the active period was positively correlated with low frequency SWA during the first two hours of the subsequent light phase (Pearson's, R^2^ = 0.49, p = 0.03), suggesting that the decrease in low frequency SWA is due in part to reduced wake during the active period. One possibility is that aged animals have less opportunity for exploration during the dark phase, which leads to reduced low frequency SWA during the early part of the light phase.

## Discussion

We confirmed and extended previous studies examining age-associated changes in sleep/wake patterns with disturbances of sleep and wakefulness in aged mice [7], [8], [9], [10], [11], [12], [13], [14], [15], [16], [17], [18]. We found that aged mice have reduced wakefulness and did not sustain long periods of wake during the active phase. Decreased wake with aging was accompanied by increased NREM sleep and aged mice did not sustain long periods of NREM sleep compared to young adult animals. Using the spike and slab formulation, we found that transitions between wakefulness and NREM sleep seem particularly sensitive to the deleterious effects of aging. Interestingly, destabilization of wakefulness and NREM sleep have also been reported in the elderly [28], [29], [30]. Spectral analysis of EEG signals revealed that activity in the theta frequency range, a correlate of arousal, was slowed in aged animals. Slow wave activity (SWA) at low frequencies (0.5–1.5 Hz) during the early part of the light phase is sensitive to vigilance and exploration during the previous period of wakefulness. We found that aged animals have decreased SWA at low frequencies, suggesting that aging also reduces vigilance during the active period. Taken together, our results suggest that decreased wakefulness in aged mice is due to the inability to sustain long periods, which causes reduced arousal and vigilance.

Studies in the past 50 years have mapped the complex neural circuitry that controls wakefulness and sleep. The consensus based on these electrophysiological, genetic and lesion experiments is that interactions between wake-producing and sleep-promoting networks control transitions from one state to the next, whereas separate components of these networks stabilize each state [27], [31], [32], [33], [34]. Consistent with this idea, behavioral states can be further classified into short and long bouts in rodents [20], [21], [22], [35], [36], [37]. Historically, the conventional measures that are used to characterize behavioral structure measure each of these components separately by computing the number of transitions from one state to the next, the number of bouts for each state and the average bout duration for wake, NREM and REM sleep [27], [38]. However, these standard metrics are correlated with one another and do not give independent views of each behavioral state. Our improved understanding of the biological mechanisms governing sleep/wake structure necessitated the creation of new models that better reflect and capture the complexity of these processes. Some recent models of sleep/wake dynamics have used a mixture of distributions to account for the uneven distribution of bout lengths [22], [23], [39], [40]. Our approach builds upon this literature, using a mixture of two distributions to simultaneously model short and long processes for each state transition. This spike and slab formulation permits the quantification of all unique states, number of bouts and bout duration for each sub-stage of wake, NREM and REM sleep. We believe this model better reflects the complexity of sleep/wake dynamics in mice. We used 4-second epochs to score sleep because it is the more commonly used sleep scoring method in mice [9], [24], [41]. The spike and slab formulation was previously used to confirm that sleep/wake architecture is under genetic control [23]. Both 4-second and 10-second epochs were used to score sleep stages in this study and the length of the scoring epoch had no bearing on the outcome of the spike and slab analysis [23]. Future work could examine whether scoring sleep/wake using shorter and longer epochs (i.e. 2 or 8 seconds) would affect the results of the spike and slab analysis in the context of aging. The current study shows for the first time that spike-and-slab analysis can be used to detect changes in sleep/wake microstructure that accompany aging in mice.

Using the spike and slab analysis, we found that transitions from wake to NREM sleep and NREM sleep to wake were uniquely altered by aging and that REM sleep architecture was unchanged with age. These results are consistent with rodent and human studies, which show that sleep becomes more fragmented with age [9], [10], [13], [28], [29], [30]. Changes in sleep architecture may contribute to alterations in the quality of sleep and wake in aged animals. In rats, hippocampal theta activity during wakefulness is indicative of arousal, exploratory behavior, and spatial navigation [42] and we found that aging slowed the peak in the theta component of the wake EEG spectra. Theta activity is also prevalent during REM sleep and aging slowed the peak in the theta component of the REM EEG spectra. Interestingly, spatial navigation is impaired by aging [11], [43], and theta activity in the hippocampus during REM sleep is experience dependent [44]. Hence, one possibility is that decreased exploration in aged mice alters hippocampal theta activity during wakefulness and during subsequent REM sleep. We measured SWA (0.5–4 Hz), a marker of sleep pressure and SWA at low frequencies (0.5–1.5 Hz) during NREM sleep because this low frequency range is particularly sensitive to changes in exploratory behavior and reductions in arousal during the previous wake period [26], [27]. For instance, animals with lesions to the LC show reduced exploratory behavior and decreased low frequency SWA during the early part of the light phase [26]. SWA (0.5–4 Hz) during the dark period was lower in aged mice, suggesting that fragmented wakefulness interfered with the normal build up of sleep pressure during the active period. Consistent with this possibility, we found that reduced wake time during the dark period likely contributes to decreased low frequency SWA (0.5–1.5 Hz) in aged mice during the following first two hours of the light phase. Because the correlation between wake time and SWA is fairly weak, other factors are likely to contribute to reduced SWA in lower frequency ranges in aged mice. Theta activity and exploratory behavior during wake can affect subsequent SWA during sleep [45], suggesting that slower TPF and lower SWA (<1.5 Hz) in aged animals may be linked. Studies in human and rodents indicate that deterioration of sleep/wake with aging may contribute to age-related cognitive impairments [46], [47], [48], [49], [50], [51], [52]. Our results suggest that aging affects the quality of wakefulness, which may also contribute to memory deficits. Further studies would be needed to explore this possibility.

The age-related changes in sleep/wake patterns that we observed are specific to circadian time, as previously reported [7], [9], [17], indicating that changes in circadian rhythms with aging may be linked to disruptions of sleep/wake states. Consistent with this hypothesis, the superchiasmatic nucleus (SCN), which drives circadian rhythm in mammals, is directly affected by aging. SCN cells show altered firing patterns or cease to fire rhythmically in aged mice [53], [54], which may lead to reduced wakefulness during the active period [53], [55]. In addition, the rhythm of clock gene expression in the SCN of aged mice shows reduced amplitude [56]. Therefore, lower SCN outputs during the dark phase may contribute to destabilization of wakefulness and NREM sleep.

In conclusion, the spike-and-slab approach, which simultaneously models short and long processes for all state transitions, is useful for characterizing age-related changes in sleep/wake microstructure. The major effect of age is to limit the durations of NREM sleep and wake that old mice can sustain during the active period. This provides a foundation for future investigations of the mechanisms involved in how the maintenance of wake and NREM sleep is altered by aging.

## References

[1] Bliwise DL (1993) Sleep in normal aging and dementia. Sleep 16: 40–81. 8456235 10.1093/sleep/16.1.40

[2] Dijk DJ, Beersma DG, van den Hoofdakker RH (1989) All night spectral analysis of EEG sleep in young adult and middle-aged male subjects. Neurobiol Aging 10: 677–682. 2628779 10.1016/0197-4580(89)90004-3

[3] Dijk DJ, Duffy JF, Czeisler CA (2000) Contribution of circadian physiology and sleep homeostasis to age-related changes in human sleep. Chronobiol Int 17: 285–311. 10841208 10.1081/cbi-100101049

[4] Ehlers CL, Kupfer DJ (1989) Effects of age on delta and REM sleep parameters. Electroencephalogr Clin Neurophysiol 72: 118–125. 2464482 10.1016/0013-4694(89)90172-7

[5] Landolt HP, Dijk DJ, Achermann P, Borbely AA (1996) Effect of age on the sleep EEG: slow-wave activity and spindle frequency activity in young and middle-aged men. Brain Res 738: 205–212. 8955514 10.1016/s0006-8993(96)00770-6

[6] Prinz PN (1995) Sleep and sleep disorders in older adults. J Clin Neurophysiol 12: 139–146. 7797628 10.1097/00004691-199503000-00004

[7] Colas D, Cespuglio R, Sarda N (2005) Sleep wake profile and EEG spectral power in young or old senescence accelerated mice. Neurobiol Aging 26: 265–273. 15582754 10.1016/j.neurobiolaging.2004.03.004

[8] Eleftheriou BE, Zolovick AJ, Elias MF (1975) Electroencephalographic changes with age in male mice. Gerontologia 21: 21–30. 166900 10.1159/000212027

[9] Hasan S, Dauvilliers Y, Mongrain V, Franken P, Tafti M (2010) Age-related changes in sleep in inbred mice are genotype dependent. Neurobiol Aging 33: 195.e13–26. 10.1016/j.neurobiolaging.2010.05.010 20619936

[10] Ingram DK, London ED, Reynolds MA (1982) Circadian rhythmicity and sleep: effects of aging in laboratory animals. Neurobiol Aging 3: 287–297. 7170046 10.1016/0197-4580(82)90017-3

[11] Markowska AL, Stone WS, Ingram DK, Reynolds J, Gold PE, et al (1989) Individual differences in aging: behavioral and neurobiological correlates. Neurobiol Aging 10: 31–43. 2569170 10.1016/s0197-4580(89)80008-9

[12] Mendelson WB, Bergmann BM (1999) Age-related changes in sleep in the rat. Sleep 22: 145–150. 10201059 10.1093/sleep/22.2.145

[13] Naidoo N, Zhu J, Zhu Y, Fenik P, Lian J, et al (2011) Endoplasmic reticulum stress in wake-active neurons progresses with aging. Aging Cell 10: 640–649. 21388495 10.1111/j.1474-9726.2011.00699.x PMC3125474

[14] Rosenberg RS, Zepelin H, Rechtschaffen A (1979) Sleep in young and old rats. J Gerontol 34: 525–532. 221565 10.1093/geronj/34.4.525

[15] Stone WS, Altman HJ, Berman RF, Caldwell DF, Kilbey MM (1989) Association of sleep parameters and memory in intact old rats and young rats with lesions in the nucleus basalis magnocellularis. Behav Neurosci 103: 755–764. 2765180 10.1037//0735-7044.103.4.755

[16] Van Gool WA, Mirmiran M (1983) Age-related changes in the sleep pattern of male adult rats. Brain Res 279: 394–398. 6640355 10.1016/0006-8993(83)90217-2

[17] Welsh DK, Richardson GS, Dement WC (1986) Effect of age on the circadian pattern of sleep and wakefulness in the mouse. J Gerontol 41: 579–586. 3745812 10.1093/geronj/41.5.579

[18] Zepelin H, Whitehead WE, Rechtschaffen A (1972) Aging and sleep in the albino rat. Behav Biol 7: 65–74. 4339671 10.1016/s0091-6773(72)80189-5

[19] Desarnaud F, Murillo-Rodriguez E, Lin L, Xu M, Gerashchenko D, et al (2004) The diurnal rhythm of hypocretin in young and old F344 rats. Sleep 27: 851–856. 15453542 10.1093/sleep/27.5.851 PMC1201560

[20] Joho RH, Marks GA, Espinosa F (2006) Kv3 potassium channels control the duration of different arousal states by distinct stochastic and clock-like mechanisms. Eur J Neurosci 23: 1567–1574. 16553620 10.1111/j.1460-9568.2006.04672.x

[21] Brightwell JJ, Gallagher M, Colombo PJ (2004) Hippocampal CREB1 but not CREB2 is decreased in aged rats with spatial memory impairments. Neurobiol Learn Mem 81: 19–26. 14670355 10.1016/j.nlm.2003.08.001

[22] Simasko SM, Mukherjee S (2009) Novel analysis of sleep patterns in rats separates periods of vigilance cycling from long-duration wake events. Behav Brain Res 196: 228–236. 18835301 10.1016/j.bbr.2008.09.003 PMC2617706

[23] McShane BB, Galante RJ, Jensen ST, Naidoo N, Pack AI, et al (2010) Characterization of the bout durations of sleep and wakefulness. J Neurosci Methods 193: 321–333. 20817037 10.1016/j.jneumeth.2010.08.024 PMC2970733

[24] Franken P, Chollet D, Tafti M (2001) The homeostatic regulation of sleep need is under genetic control. J Neurosci 21: 2610–2621. 11306614 10.1523/JNEUROSCI.21-08-02610.2001 PMC6762509

[25] Borbely AA, Baumann F, Brandeis D, Strauch I, Lehmann D (1981) Sleep deprivation: effect on sleep stages and EEG power density in man. Electroencephalogr Clin Neurophysiol 51: 483–495. 6165548 10.1016/0013-4694(81)90225-x

[26] Cirelli C, Huber R, Gopalakrishnan A, Southard TL, Tononi G (2005) Locus ceruleus control of slow-wave homeostasis. J Neurosci 25: 4503–4511. 15872097 10.1523/JNEUROSCI.4845-04.2005 PMC6725032

[27] Qu WM, Xu XH, Yan MM, Wang YQ, Urade Y, et al (2010) Essential role of dopamine D2 receptor in the maintenance of wakefulness, but not in homeostatic regulation of sleep, in mice. J Neurosci 30: 4382–4389. 20335474 10.1523/JNEUROSCI.4936-09.2010 PMC6634511

[28] Ancoli-Israel S (2009) Sleep and its disorders in aging populations. Sleep medicine 10 (Suppl 1) S7–11. 19647483 10.1016/j.sleep.2009.07.004

[29] Cooke JR, Ancoli-Israel S (2011) Normal and abnormal sleep in the elderly. Handbook of clinical neurology 98: 653–665. 21056216 10.1016/B978-0-444-52006-7.00041-1 PMC3142094

[30] Kryger M, Monjan A, Bliwise D, Ancoli-Israel S (2004) Sleep, health, and aging. Bridging the gap between science and clinical practice. Geriatrics 59: 24–26, 29–30. 14755865

[31] Lu J, Sherman D, Devor M, Saper CB (2006) A putative flip-flop switch for control of REM sleep. Nature 441: 589–594. 16688184 10.1038/nature04767

[32] Qiu MH, Vetrivelan R, Fuller PM, Lu J (2010) Basal ganglia control of sleep-wake behavior and cortical activation. Eur J Neurosci 31: 499–507. 20105243 10.1111/j.1460-9568.2009.07062.x PMC3928571

[33] Saper CB, Fuller PM, Pedersen NP, Lu J, Scammell TE (2011) Sleep state switching. Neuron 68: 1023–1042. 10.1016/j.neuron.2010.11.032 PMC3026325 21172606

[34] Takahashi K, Kayama Y, Lin JS, Sakai K (2010) Locus coeruleus neuronal activity during the sleep-waking cycle in mice. Neuroscience 169: 1115–1126. 20542093 10.1016/j.neuroscience.2010.06.009

[35] Deboer T, Tobler I (1996) Shortening of the photoperiod affects sleep distribution, EEG and cortical temperature in the Djungarian hamster. Journal of comparative physiology A, Sensory, neural, and behavioral physiology 179: 483–492. 10.1007/BF00192315 8828178

[36] Franken P, Tobler I, Borbely AA (1992) Cortical temperature and EEG slow-wave activity in the rat: analysis of vigilance state related changes. Pflugers Archiv : European journal of physiology 420: 500–507. 1614823 10.1007/BF00374625

[37] Mochizuki T, Crocker A, McCormack S, Yanagisawa M, Sakurai T, et al (2004) Behavioral state instability in orexin knock-out mice. J Neurosci 24: 6291–6300. 15254084 10.1523/JNEUROSCI.0586-04.2004 PMC6729542

[38] Ouyang M, Hellman K, Abel T, Thomas SA (2004) Adrenergic signaling plays a critical role in the maintenance of waking and in the regulation of REM sleep. J Neurophysiol 92: 2071–2082. 15190089 10.1152/jn.00226.2004

[39] Behn CG, Brown EN, Scammell TE, Kopell NJ (2007) Mathematical model of network dynamics governing mouse sleep-wake behavior. J Neurophysiol 97: 3828–3840. 17409167 10.1152/jn.01184.2006 PMC2259448

[40] Chu-Shore J, Westover MB, Bianchi MT (2010) Power law versus exponential state transition dynamics: application to sleep-wake architecture. PLoS One 5: e14204. 21151998 10.1371/journal.pone.0014204 PMC2996311

[41] Franken P, Malafosse A, Tafti M (1998) Genetic variation in EEG activity during sleep in inbred mice. Am J Physiol 275: R1127–1137. 9756543 10.1152/ajpregu.1998.275.4.R1127

[42] Wyble BP, Hyman JM, Rossi CA, Hasselmo ME (2004) Analysis of theta power in hippocampal EEG during bar pressing and running behavior in rats during distinct behavioral contexts. Hippocampus 14: 662–674. 15301442 10.1002/hipo.20012

[43] Barnes CA (1979) Memory deficits associated with senescence: a neurophysiological and behavioral study in the rat. J Comp Physiol Psychol 93: 74–104. 221551 10.1037/h0077579

[44] Poe GR, Nitz DA, McNaughton BL, Barnes CA (2000) Experience-dependent phase-reversal of hippocampal neuron firing during REM sleep. Brain research 855: 176–180. 10650147 10.1016/s0006-8993(99)02310-0

[45] Huber R, Tononi G, Cirelli C (2007) Exploratory behavior, cortical BDNF expression, and sleep homeostasis. Sleep 30: 129–139. 17326538 10.1093/sleep/30.2.129

[46] Altena E, Ramautar JR, Van Der Werf YD, Van Someren EJ (2010) Do sleep complaints contribute to age-related cognitive decline? Progress in brain research 185: 181–205. 21075240 10.1016/B978-0-444-53702-7.00011-7

[47] Fillit HM, Butler RN, O'Connell AW, Albert MS, Birren JE, et al (2002) Achieving and maintaining cognitive vitality with aging. Mayo Clinic proceedings Mayo Clinic 77: 681–696. 10.4065/77.7.681 12108606

[48] Mander BA, Rao V, Lu B, Saletin JM, Lindquist JR, et al (2013) Prefrontal atrophy, disrupted NREM slow waves and impaired hippocampal-dependent memory in aging. Nature neuroscience 16: 357–364. 23354332 10.1038/nn.3324 PMC4286370

[49] Gerrard JL, Burke SN, McNaughton BL, Barnes CA (2008) Sequence reactivation in the hippocampus is impaired in aged rats. The Journal of neuroscience : the official journal of the Society for Neuroscience 28: 7883–7890. 18667620 10.1523/JNEUROSCI.1265-08.2008 PMC2703197

[50] Shen J, Barnes CA, McNaughton BL, Skaggs WE, Weaver KL (1997) The effect of aging on experience-dependent plasticity of hippocampal place cells. The Journal of neuroscience : the official journal of the Society for Neuroscience 17: 6769–6782. 9254688 10.1523/JNEUROSCI.17-17-06769.1997 PMC6573142

[51] Pang KC, Miller JP, Fortress A, McAuley JD (2006) Age-related disruptions of circadian rhythm and memory in the senescence-accelerated mouse (SAMP8). Age 28: 283–296. 22253495 10.1007/s11357-006-9013-9 PMC3259149

[52] Stone WS (1989) Sleep and aging in animals. Relationships with circadian rhythms and memory. Clinics in geriatric medicine 5: 363–379. 2665917

[53] Farajnia S, Michel S, Deboer T, vanderLeest HT, Houben T, et al (2012) Evidence for neuronal desynchrony in the aged suprachiasmatic nucleus clock. The Journal of neuroscience : the official journal of the Society for Neuroscience 32: 5891–5899. 22539850 10.1523/JNEUROSCI.0469-12.2012 PMC6703600

[54] Nygard M, Hill RH, Wikstrom MA, Kristensson K (2005) Age-related changes in electrophysiological properties of the mouse suprachiasmatic nucleus in vitro. Brain Res Bull 65: 149–154. 15763181 10.1016/j.brainresbull.2004.12.006

[55] Nakamura TJ, Nakamura W, Yamazaki S, Kudo T, Cutler T, et al (2011) Age-related decline in circadian output. The Journal of neuroscience : the official journal of the Society for Neuroscience 31: 10201–10205. 21752996 10.1523/JNEUROSCI.0451-11.2011 PMC3155746

[56] Weinert H, Weinert D, Schurov I, Maywood ES, Hastings MH (2001) Impaired expression of the mPer2 circadian clock gene in the suprachiasmatic nuclei of aging mice. Chronobiol Int 18: 559–565. 11475423 10.1081/cbi-100103976

